# Coronavirus epidemic and geriatric mental healthcare in China: how a coordinated response by professional organizations helped older adults during an unprecedented crisis

**DOI:** 10.1017/S1041610220000551

**Published:** 2020-04-09

**Authors:** Huali Wang, Tao Li, Serge Gauthier, Enyan Yu, Yanqing Tang, Paola Barbarino, Xin Yu

**Affiliations:** 1 Dementia Care and Research Center, Peking University Institute of Mental Health (Sixth Hospital), Beijing Dementia Key Lab, Beijing 100191, China; 2 NHC Key Laboratory of Mental Health, National Clinical Research Center for Mental Disorders (Peking University), Beijing 100191, China; 3 McGill Center for Studies in Aging, Douglas Mental Health Research Institute, McGill University, Montreal, Canada; 4 Department of Psychological Medicine, Cancer Hospital of the University of Chinese Academy of Sciences, Zhejiang Cancer Hospital, Hangzhou 310022, China; 5 Department of Psychiatry, the First Hospital of China Medical University, Shenyang 110001, China; 6 Alzheimer’s Disease International, London SE1 0BL, UK

As of March 2020, more than one-third of confirmed cases with the 2019 coronavirus disease (COVID-19) in China were aged 60 years and above, and more than 80% of deaths due to COVID-19 occurred in older adults (The Novel Coronavirus Pneumonia Emergency Response Epidemiology Team and Chinese Center for Disease Control and Prevention, 2020). When exposed to disaster or emergency, older people are vulnerable to developing mental health and behavioral issues, such as becoming more anxious, stressed, agitated, and having sleep problems (Jia *et al.*, 2010).

During these unprecedented times, Chinese professional organizations brought together geriatric mental healthcare teams of diverse disciplines and coordinated psychosocial support for older adults and their caregivers affected by the coronavirus epidemic. Learning lessons from China may empower the world to address the mental health needs of older people in a timely manner during the COVID-19 pandemic and emergency crisis.

## Why Old Adults Became More Vulnerable

In the Chinese example, there was a convergence of many factors that led to the development of mental health and behavioral problems.

First, older adults had low awareness and limited access to accurate information and facts about the COVID-19 outbreak. This resulted in either excessive worries or in obliviousness to the warnings. Second, older adults attended family gatherings during the Chinese New Year. Such types of social activities increased their risk of being exposed to virus transmission which then resulted in quarantine and isolation in case of suspected or confirmed cases of the virus. Third, the strict security measures in the community prevented outdoor social activities, such as square dancing, park exercises, and parties. Fourth, the extended lock-down rules in nursing homes blocked face-to-face communications between older adults and their families (Ministry of Civil Affairs of China, 2020). Last, but not least, some older adults who lived alone or did not have close relatives lost their domestic helpers who were prohibited from traveling, or were in quarantine themselves, upon returning from lockdown areas.

Because of the aforementioned, the social participation and engagement of older adults were drastically reduced.

Just as in the general population, some older adults showed signs of fear and anxiety; some withdrew from group activities and became less motivated; and others ignored the warning messages and refused to take self-quarantine measures, such as wearing masks (Wang *et al*., 2020).

Those with preexisting mental health issues may have specific clinical features. For example, people with psychosis may become overly suspicious while in quarantine; people with depression and anxiety may feel more stressed and depressed; people with hypochondria are likely to have more physical concerns; people with cognitive decline or dementia may present more challenging behaviors. They could not get access to care support as usual due to social distancing. In addition, limited access to clinical services may have reduced compliance to prescribed medications of those affected by mental disorders (Yang *et al.*, 2020). Therefore, the social consequence of the COVID-19 outbreak may put older people with preexisting mental disorders at risk of relapse.

## How Professional Organizations Collaborated to Develop a National Level Response

During the COVID-19 outbreak, suspected and confirmed cases could receive mental health care in hospitals and mobile cabin hospitals in Wuhan with no regard for age, following the guidance of National Health Commission of China (NHC) (National Health Commission of China, 2020; Bao *et al.*, 2020; Ma *et al*., 2020). Mental health professionals joined the emergency medical teams so that psychological first aid could be provided immediately. The Chinese Society of Geriatric Psychiatry, in collaboration with the Chinese Society of Psychiatry, the Chinese Psychiatrist Association, and the Chinese Association for Mental Health, responded to the crisis promptly with an interdisciplinary solution. Mental health professionals, social workers, nursing home administrators, and volunteers delivered mental health and psychosocial support (MHPSS) for older adults collaboratively, especially for community-dwelling residents and nursing-home residents. Within a team, geriatric mental health professionals took the lead and supported the team members coming from other disciplines. The competences and the roles of each type of health professionals during the COVID-19 outbreak were as follows:

First, the multidisciplinary experts developed a psychosocial self-help guidebook as support for older adults. The book, both in printed and audible format, was offered free online (Wang *et al*., 2020). In addition, the five societies mentioned above developed expert recommendations on MHPSS for persons with cognitive disorders (Chinese Society of Geriatric Psychiatry *et al.*, 2020). These documents served as a guide to senior citizens, their caregivers, as well as professionals.

Second, social workers, working closely with community administrators, took primary responsibility for supporting and providing a safe environment for senior citizens (National Health Commission of China and Ministry of Civil Affairs of China, 2020; Wang *et al*., 2020). For example, social workers would seek supplies of masks and hand sanitizers for any senior citizen who had run out of these supplies. Social workers also helped community residents to learn how to shop online, how to make an appointment for medical care, and how to do relaxation training at home. Whenever an older adult’s mental status was deteriorating, the social workers liaised with a geriatric psychiatrist for further consultation or made a referral to a clinic.

Third, psychological counselors were more involved in online consultation or in psychological counseling through telephone hotlines (Liu *et al.*, 2020). The online platform overcame the geographic barriers, and people could get access to the support wherever they lived. When an older adult was at higher risk of physical threat or mental health problems, psychological first aid was initiated by a local social worker under the online supervision of psychological counselors (World Health Organization *et al.*, 2014).

Fourth, public education through social media (e.g. WeChat, ByteDance) became the most common form of communication. Online courses on relaxation training, music, art, and psychomotor training were delivered through this medium. Older adults could get access to the classes with the help of their family members.

Last, but not least, in response to staff burnout experiences and anxiety in nursing homes, psychological counselors provided support to nursing homes through online consultations and webinars.

Observing the positive impact of the MHPSS on persons with cognitive disorders and their caregivers, Alzheimer’s Disease International (ADI) endorsed the Chinese expert recommendations and shared the Chinese experience with other ADI members (Wang *et* *al*., 2020). The Inter-Agency Standing Committee (IASC) of the United Nations updated the MHPSS guideline with specific interventions for older adults (Inter-Agency Standing Committee, 2007; 2020). As the COVID-19 outbreak threatens more and more countries (World Health Organization, 2020), older adults in the world are facing similar challenges. The IASC guideline will serve the whole world.

## Lessons for the Future

In Chinese, crisis (“危机, *weiji*”) is the combination of danger (危, *wei*) and opportunity (机, *ji*). Undoubtedly, the outbreak of COVID-19 has caused a significant impact in the life of senior citizens. However, it has also created opportunities to improve mental health care for older people exposed to stress in emergency situation in different ways.

First, it raised awareness of the importance of establishing a mechanism of emergency response to provide MHPSS in society. There has never been a specific MHPSS guideline in response to disaster or crisis for older adults, not to mention for persons with cognitive disorders. The published self-help guidance on psychological support and expert recommendations for persons with dementia now demonstrates the preparedness of MHPSS in disaster management (Chinese Society of Geriatric Psychiatry *et al.*, 2020; Wang *et al*., 2020). The inclusion of specific interventions for older people in the IASC guideline will become a new standard in this field (Inter-Agency Standing Committee, 2020). We could envision how the psychological distress of older people would be immediately addressed in a future emergency.

Second, the urge to implement strict community quarantine regulation provided younger senior citizens with an opportunity to serve the community. While volunteering, they received sufficient social engagement. In addition, the volunteer work not only highlighted the importance of values such as altruism but also made the volunteer feel valued and respected in their community, which in turn, resulted in augmented resilience to stress (Richaud and Amin, 2019).

Third, flexible working time allows family members to have more frequent contact or more time to stay together and thus strengthens the family bond and support. Duty, familial obligation, and reciprocity are core beliefs for caregiving in Chinese culture (Xiong *et al.*, 2011). The time staying together with parents develops valuable memories for children. As a consequence, family bonds and the overall atmosphere within families might have improved.

Overall, during the COVID-19 outbreak, China implemented MHPSS for senior citizens in a timely manner. The local practice has boosted the global response to set a precedent for the MHPSS specific for senior citizens. Just as any coin has two sides, despite its devastating threat to global population health, the COVID-19 outbreak has also created an opportunity for building a coordinated MHPSS team, through efforts of the society, community, and family, for senior citizens in emergency or disaster settings in China and hopefully worldwide. Only through the solidarity of the mental healthcare community, can we address the specific stress-related challenges of older people during unprecedented circumstances.
